# Minicraniotomy for Standard Temporal Lobectomy: A Minimally Invasive Surgical Approach

**DOI:** 10.1155/2014/532523

**Published:** 2014-02-06

**Authors:** Faisal Al-Otaibi, Monirah Albloushi, Saleh Baeesa

**Affiliations:** ^1^Division of Neurosurgery, Neuroscience Department, King Faisal Specialist Hospital and Research Center, P.O. Box 3354, Riyadh 11211, Saudi Arabia; ^2^College of Nursing, King Saud University, Riyadh, Saudi Arabia; ^3^Division of Neurosurgery, King Abdulaziz University, Jeddah, Saudi Arabia

## Abstract

*Introduction.* The common surgical approach for standard temporal lobectomy is a question-mark skin incision and a frontotemporal craniotomy. Herein, we describe minicraniotomy approach through a linear skin incision for standard temporal lobectomy. *Methods.* A retrospective observational cohort study was conducted for a group of consecutive 21 adult patients (group I) who underwent minicraniotomy for standard temporal lobectomy utilizing a linear skin incision. This group was compared to a consecutive 17 adult patients (group II) who previously underwent a reverse question-mark skin incision and standard frontotemporal craniotomy. *Results.* The mean age was 29 and 23 for groups I and II, respectively. The mean estimated blood loss was 190 mL and 280 mL in groups I and II, respectively (*P* = 0.019). Three patients in group II developed chronic postcraniotomy headache compared to none in group I. Cosmetic outcome was excellent in group I while 4 patients in group II developed disfiguring depression at lateral sphenoid wing and anterior temple. In group I 17 out of 21 became seizure-free at one-year followup. *Conclusion.* Minicraniotomy through a linear skin incision is a sufficient surgical approach for effective standard temporal lobectomy and it has an excellent cosmetic outcome.

## 1. Introduction

Several modifications have been made to the surgical techniques and methods used to treat temporal lobe epilepsy over the last 50 years [1–4]. Performing a standard anterior temporal lobectomy consists of resecting the lateral temporal and mesial temporal structures, either en bloc or separately as popularized by Penfield [1]. The anteromedial temporal resection technique was developed by Spencer to preserve lateral temporal cortex function and to access the mesial temporal structures through the temporal pole corridor [5]. Both procedures are done traditionally through a question-mark skin incision and frontotemporal craniotomy [6]. Conversely, selective transcortical amygdalohippocampectomy is done mainly through a smaller question-mark or vertical temporal skin incision and temporal minicraniotomy [7]. A minimally invasive neurosurgical approach has shown a benefit in reducing morbidity and producing better cosmetic results [8–10]. Several modifications to temporal lobe resective surgery have been based either on resection of the epileptogenic zone, assisted by the use of electrocorticography and cortical mapping to avoid functional deficits, or on resection of the seizure onset zone, as with selective amygdalohippocampectomy. In this cohort study, we report a minimally invasive surgical technique for standard temporal lobectomy and its outcome.

## 2. Methods

### 2.1. Study Design

A total of 21 consecutive patients (group I) who had undergone a modified minimally invasive surgical approach for standard temporal lobectomy (from March 2011 till December 2012) were retrospectively analyzed. This group of patients was compared to a group of 17 consecutive patients (group II) who had undergone conventional reverse question-mark skin incision and standard frontotemporal craniotomy for temporal lobectomy. Institutional review board approval was obtained for the retrospective review of consecutive patient records pertinent to the study. The following variables were evaluated in both groups: surgery time, estimated blood loss, extent of resection, chronic postcraniotomy pain (persistent headache at one-year followup), cosmetic effect, seizure outcome, and surgical complications. Since this study is a descriptive of a surgical approach technique, the adverse effect of surgery such as the effect on memory and visual field functions was not included in this study. The extent of resection of the temporal neocortex and mesial temporal structures was measured by follow-up magnetic resonance imaging (MRI). In group I, the follow-up MRI result was compared to the intraoperative neuronavigation image guidance estimate of resection. The follow-up period was a minimum of one year (range 1–7 years).

### 2.2. Minicraniotomy Surgical Technique for Standard Temporal Lobectomy

**Figure 1:**
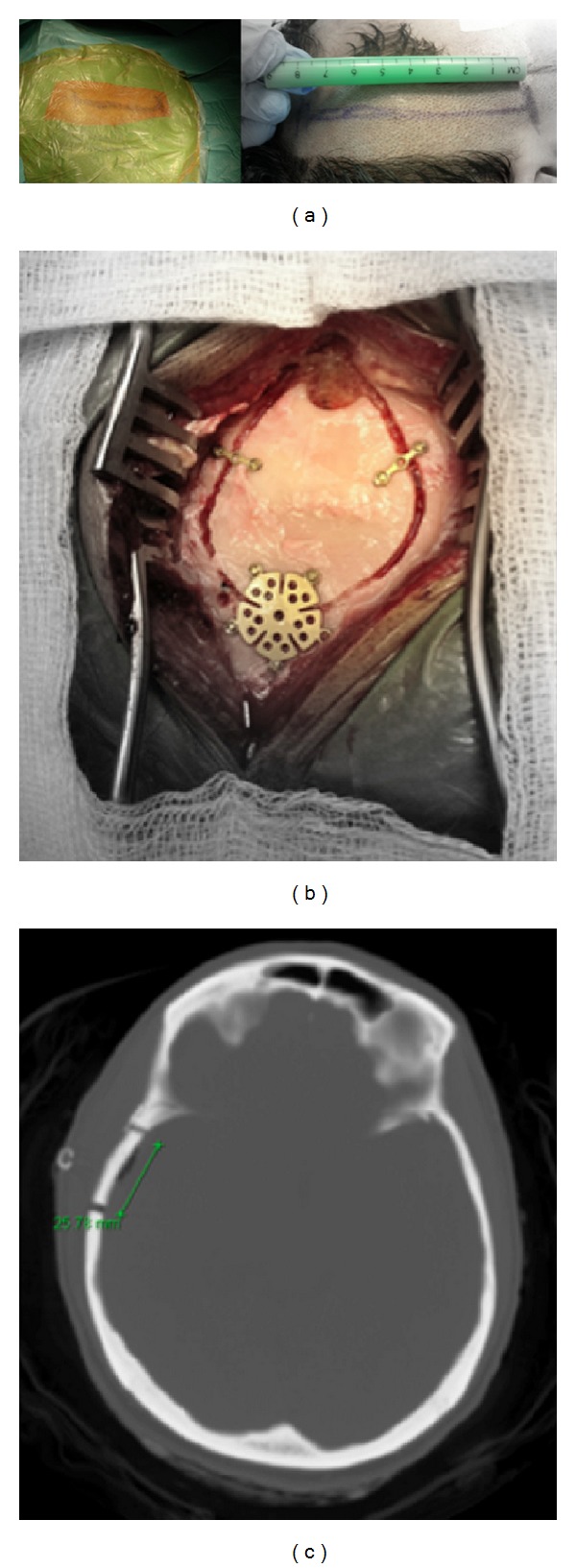
Intraoperative photographs depicting the linear temporal skin incision and minicraniotomy that was utilized in group I (a) and (b). Postoperative computerized tomography of the brain showing the size of minicraniotomy (c).

In this section, we describe the linear skin incision and minicraniotomy surgical approach for standard temporal lobectomy, Figure 1. The procedure is usually performed with the patient in the supine position, elevating the ipsilateral shoulder with a roll and rotating the head to the contralateral side. The head is tilted slightly laterally to place the zygoma at an approximately 10-degree angle from the horizontal plane of the surgical floor. Neuronavigation is applied for image guidance throughout the procedure. To avoid injury to the frontalis branch of the facial nerve, the linear skin incision is begun 1 cm above the zygoma and 1 cm anterior to the tragus and extended vertically 6–8 cm to approximately the level of superior temporal line. The superficial temporal artery is dissected and preserved as much as possible. Sharp dissection is used to open temporalis muscle and electrocautery is avoided as much as possible to minimize the subsequent atrophy of the muscle. Minicraniotomy at the temporal area is carried out with size of 3 cm using a fast drill and one or two burr holes, Figure 1. The upper edge of the minicraniotomy is at the sylvian fissure level guided by neuronavigation. A U-shaped durotomy incision is performed with the base reflected anteriorly. The posterior extent of neocortical resection is guided by neuronavigation and direct measurement from the temporal tip at the level of the middle temporal gyrus. The posterior resection line is placed at 4 cm on the nondominant temporal lobe and 3 cm on the dominant temporal lobe at the level of the middle temporal gyrus. The posterior resection is slanted anteriorly across the superior temporal gyrus to avoid the primary auditory cortex. More posterior resection is usually done at the inferior temporal gyrus and less resection at the superior temporal gyrus.

The microscope is then introduced to the surgical field, and a subpial dissection is done at the superior margin of the superior temporal gyrus using an ultrasonic aspirator exposing the sylvian fissure down to the insular cortex. The insula is exposed, and dissection extending to the lateral uncus is performed. The temporal pole is reflected laterally after the coagulation and division of the anterior leptomeninges. The posterior resection line is extended from the superior gyrus through the middle gyrus and into the inferior temporal gyrus. This line is then extended medially through the fusiform gyrus to the collateral sulcus. The temporal horn is entered through the white matter above the fusiform gyrus. The wall of the temporal horn can be identified by the bluish ependyma. Subsequently, opening of the ventricle anteriorly exposes the hippocampal head. The temporal stem is resected at the inferior circular sulcus. The temporal neocortex is removed by dividing the basal leptomeninges lateral to the temporal horn exposure.

**Figure 2:**
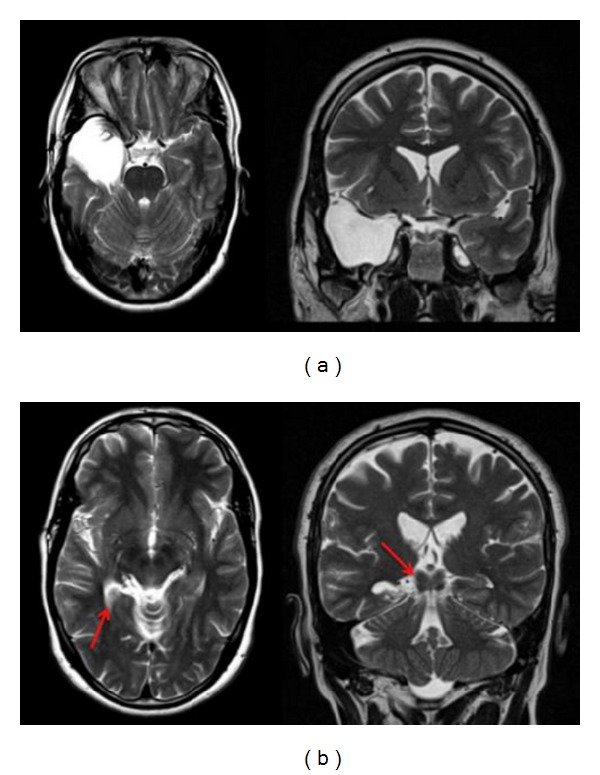
Postoperative MRI brain (axial and coronal T2 weighted images) showing the extent of neocortex (a) and posterior hippocampus resection (b). Note: the posterior resection beyond the level of brainstem folliculi (quadrigeminal plate level) (see red arrow).

**Figure 3:**
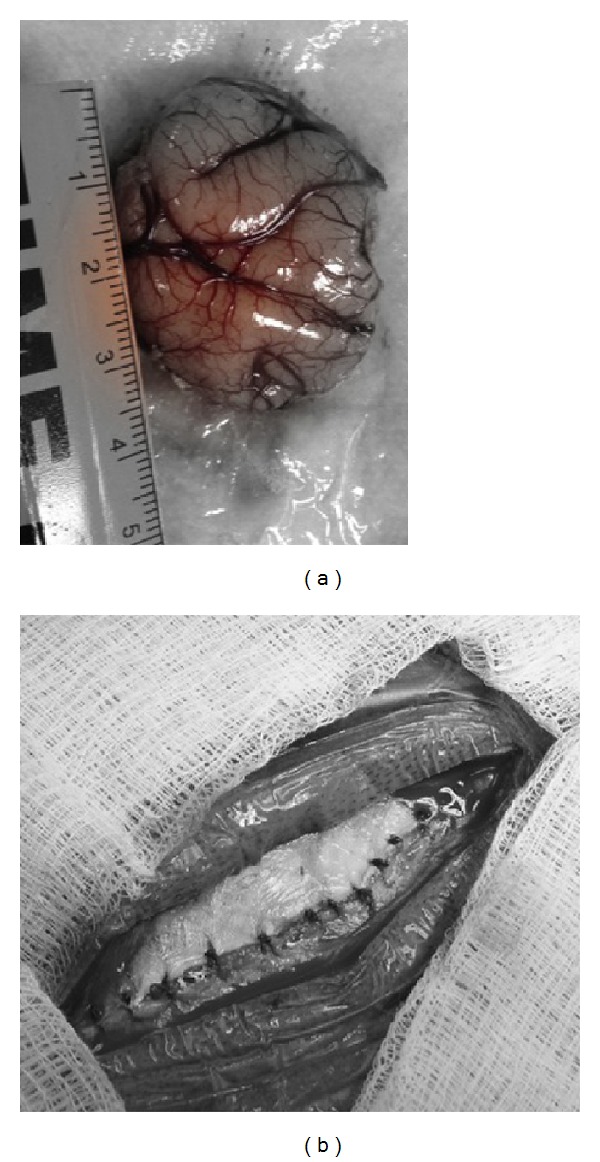
Intraoperative photos demonstrating neocortex specimen (a) and temporalis muscle fascia closure (b).

The mesial temporal structures are resected using an ultrasonic aspirator from anterior to posterior. No retraction is used during this step. In our practice, the posterior portion of the hippocampus is removed using an ultrasonic aspirator to the level of the midbrain tectum, as identified by image guidance, Figure 2. Next, homeostasis is secured, and wound closure is performed in a standard manner, Figure 3.

### 2.3. Statistical Analysis

Data were entered and analyzed using the SPSS 17th edition (Chicago, IL, USA). The nonparametric Mann-Whitney test was used to compare differences between estimated blood loss in both groups. Statistic significance was determined if *P* value <0.05.

## 3. Results

The mean age was 29.2 years in group I and 23.6 years in group II. The surgical opening average time from skin to dura was 20 minutes, and the closure average time from dura to skin was about 30 minutes in group I, Table 1. The opening and closure times were not calculated for group II. The overall operative time from skin to skin was on average 3 hours and 20 minutes in group I and 3 hours and 40 minutes in group II. The mean estimated blood loss was 190 mL and 280 mL in groups I and II, respectively (*P*  value = 0.019). The average length of hospital stay was 4 days for group I and 4.5 days for group II. The cosmetic result was excellent in group I with no presence of wide scar formation. In group II, four patients had a disfiguring depression at the anterior temporal area. Chronic postcraniotomy pain at the surgical site occurred in three patients in group II and none of the patients in group I. Out of those three patients, one had continuous local pain at the surgical site and two had chronic intermittent hemicranial headaches.

**Figure 4:**
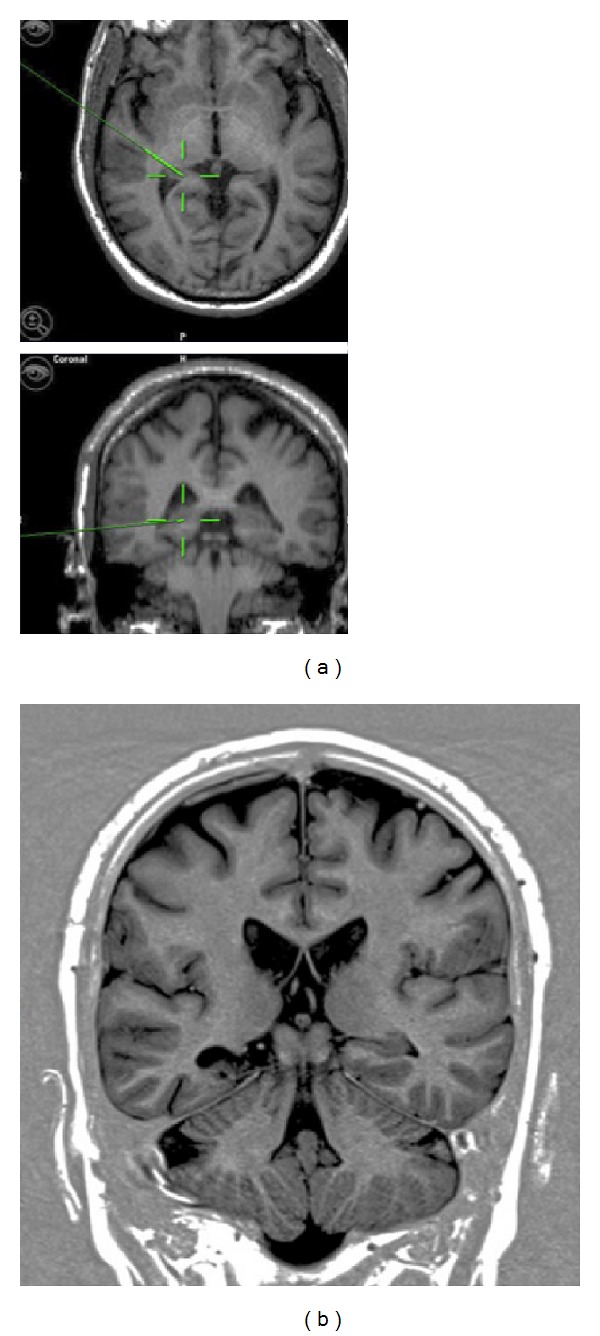
Intraoperative neuronavigation (a) and postoperative MRI brain (b) showing the difference in posterior extent of hippocampus resection. Intraoperative neuronavigation image guidance does overestimate the extent of posterior resection due to intraoperative brain shift.

**Figure 5:**
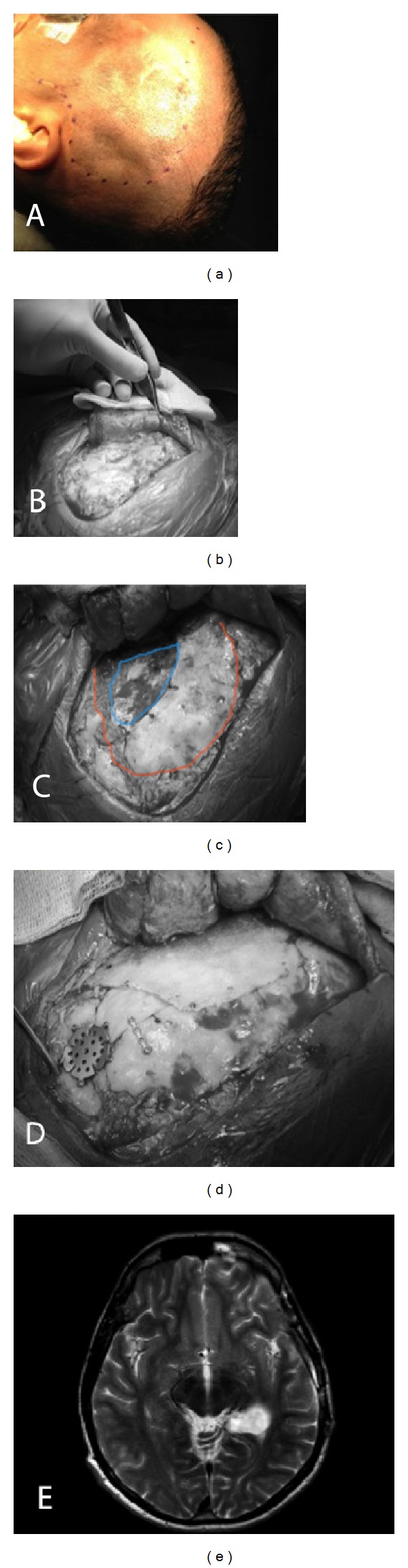
Case illustration: a 34-year-old man who was previously operated on for left mesial temporal lesion and epilepsy using large left frontotemporal craniotomy. He underwent a redo lesionectomy and temporal lobectomy using smaller craniotomy access. Large frontotemporal reverse question-mark skin incision is demonstrated in the intraoperative photo (a). Exposure of bone depicting the irregularity of bone surface (b). Small craniotomy access is demonstrated as compared to the previously performed craniotomy (c). Reconstruction of lateral sphenoid wing and bone depressions for better cosmetic result (d). The extent of mesial temporal resection is depicted in a follow-up MRI brain (e).

The extent of resection of the temporal neocortex and mesial structures measured on follow-up MRI was almost similar in both groups. The extent of posterior hippocampus resection was found to be at the level of the quadrigeminal plate or posterior to it, Figure 2. Intraoperative neuronavigation image guidance was found to overestimate the extent of hippocampus posterior resection by an average of 16 mm, Figure 4. In terms of seizure freedom, there was no significant difference between the groups. In group I, 17 out of 21 patients (80.9%) became seizure-free at followup of one year or more. In group II, 12 out of 17 patients (70.6%) became seizure-free, taking into consideration that bitemporal epilepsy was found in three patients in group II and only two patients in group I. Complications included one patient with a superficial wound infection and one with transient third nerve partial palsy in group II. In group I, one patient developed transient limitation of mouth opening ability associated with intermittent pain at the temporomandibular joint area, and one patient developed a small focal hair loss posterior to the wound, which was related to skin retraction that subsided after six-month followup.  Table 2 summarizes different variables in the two patient groups.  Figure 5 demonstrates a case illustration of a redo temporal lobectomy and lesionectomy utilizing small access craniotomy and comparing that to the previously used craniotomy access.

## 4. Discussion

Minimally invasive surgical approaches are widely used by neurosurgeons. The main advantages are shorter operative times, less surgical trauma, shorter hospitalization times, less chance of postoperative pain, and achieving of excellent cosmetic results [11]. Surgical approaches for temporal lobectomy to treat medically intractable epilepsy have several technical variations. These include standard temporal lobectomy, anteromedial temporal lobectomy, selective amygdalohippocampectomy, and stereotactic approaches [2, 5, 12, 13]. Penfield popularized standard temporal lobectomy using a large reverse question-mark skin incision and frontotemporal craniotomy and bone removal of the lateral sphenoid wing for better exposure [1]. He utilized intraoperative electrocorticography and cortical mapping in the temporal lobectomy procedure, which led him to better exposure of the temporal lobe. Many neurosurgeons have adapted Penfield surgical techniques with minimal variations. This technique has been named “standard” temporal lobectomy because it is a reproducible procedure and varies little from surgeon to surgeon. Most published series used a frontotemporal reverse question-mark skin incision followed by frontotemporal craniotomy with lateral sphenoid wing removal [14]. The extent of neocortical resection ranges between 3.5 and 6 cm, with less resection on the dominant temporal lobe. The posterior resection extent of the mesial temporal structures varies in the literature from the body of hippocampus to the most posterior hippocampus tail at the level of the quadrigmeinal plate. A recent randomized trial has demonstrated a correlation between the more posterior extent of hippocampus resection and Engel class I outcome [15]. Spencer described the anteromedial temporal lobectomy technique with limited neocortical resection to the anterior 2.5 to 3 cm of the middle and inferior temporal gyri sparing the superior gyrus [5]. In the Spencer approach, a reverse question-mark skin incision and smaller size craniotomy were used [7]. A recent meta-analysis study showed that standard temporal lobectomy confers more chance of seizure freedom as compared to selective amygdalohippocampectomy [16].

In this observational study, a minicraniotomy was utilized through a linear skin incision at the temporal area as an access for standard temporal lobectomy. The linear skin incision provides direct and faster access to the temporal bone. In addition, it provides a greater chance to preserve the superficial temporal artery, and this minimizes the incidence of postoperative temporalis muscle atrophy. In the 21 patients in group I, this approach minimized opening time as compared to performing a large reverse question-mark incision. Although this was observed by the operating team, the opening time for the comparison group was not recorded, making it difficult to draw a conclusion. No significant adverse effect was found from this linear skin incision apart from transient painless limitation of mouth opening ability, which was likely related to fibrosis formation at the temporalis muscle. Another patient in this group developed focal scalp alopecia posterior to the wound and above the ear pinna. This was likely related to the self-retaining retractor that was placed on the skin instead of on the temporalis muscle. To prevent this adverse effect, the self-retaining retractor is placed under the temporalis muscle. However, this problem resolved during subsequent followup. Minicraniotomy with no bone removal at the lateral sphenoid wing also reduces the surgical approach time and improves cosmetic effect. Moreover, it does eliminate the use of reconstruction of the bone defect.

Neocortical resection was carried out under a surgical microscope in group I, which probably increased the resection time as compared to group II in which the neocortex was removed under surgical loop. In both groups, the same surgical steps were used during neocortical resection. This technique was adapted from London Ontario group based on the surgeon's training background. The extent of neocortical resection in group I was comparable to that of group II and also was within the range of neocortical resections that have been published in the literature [14]. Therefore, the smaller bony access did not limit temporal neocortex resection. The early use of a surgical microscope after durotomy gives optimal visualization of the temporal lobe from the sylvian fissure to the base. Subpial dissection of the superior temporal gyrus off the sylvian fissure allows drainage of cerebrospinal fluid and early relaxation of the brain.

The extent of posterior hippocampus resection is standardized in our institutions at the level or posterior to the quadrigeminal plate using the cistern and tentorial curve to the hiatus as anatomical landmarks as well as the use of image guidance neuronavigation. There was no difference between the two groups regarding the extent of posterior hippocampus resection. Due to the limited number of patients, complication rates cannot be optimally compared between the two groups. The noticeable benefit of this minimally invasive approach is the optimal cosmetic result and a reduced chance of chronic postcraniotomy pain and headache. The mechanism of postcraniotomy pain in group II was likely induced by the exposed dura adhesion to temporalis muscle that may cause a stretch of the dura during jaw movement. The observed reduction in blood loss in group I is likely due to smaller skin incisions and less bone removal. The overall seizure outcome was comparable in both groups, which means that the minicraniotomy approach is as effective as the conventional surgical approach. Although this study described the effectiveness of minicraniotomy as a minimally invasive approach for standard temporal lobectomy, the small number of patients is one of the notable limitations. Moreover, this technique limits the application of brain mapping using direct electrical stimulation.

## 5. Conclusion

Minicraniotomy access through a linear skin incision for standard temporal lobectomy is a minimally invasive surgical approach that has an excellent cosmetic result and is an effective technique for temporal lobe epilepsy treatment.

## Figures and Tables

**Table 1:** Summary of group I patient data.

No.	Age Sex	Pathology	Surgery side	Extent of neocortical resection from temporal pole^]^	Extent of posterior hippocampus resection	Cosmetic result	Seizure outcome. (Engel classification)	Surgical complications
1	27 F	MTS^*ϕ*^	Rt	4 cm	QP level^Δ^	+	Class I	None
2	31 M	Normal	Rt	4 cm	QP level	+	Class II	None
3	26 M	MTS	Rt	4.5 cm	Post. to QP	+	Class I	None
4	42 M	Cavernoma	Rt	4 cm	Post. to QP	+	Class I	None
5	17 F	MTS	Lt	3.5 cm	QP level	+	Class I	None
6	20 F	MTS	Lt	3 cm	QP level	+	Class I	None
7	38 M	MTS	Rt	4 cm	QP level	+	Class I	Mouth opening limitation
8	22 F	Ganglioglioma	Lt	4 cm	Post. to QP	+	Class I	None
9	18 F	MTS	Rt	4 cm	Post. to QP	+	Class I	None
10	32 F	MTS	Rt	4.5 cm	QP level	+	Class I	None
11	24 F	MTS	Lt	3.7 cm	Post. to QP	+	Class I	None
12	44 M	MTS	Lt	3.5 cm	QP level	+	Class I	None
13	21 M	MTS	Rt	3.8 cm	Post. to QP	+	Class II	None
14	28 F	MTS	Rt	4 cm	QP level	+	Class I	None
15	18 F	MTS	Lt	3.5 cm	QP level	+	Class I	None
16	29 M	Normal	Rt	3.8 cm	QP level	+	Class I	Focal alopecia
17	31 F	Ganglioglioma and MTS	Lt	3.6 cm	Post. to QP	+	Class I	None
18	27 F	Neocortical astrogliosis	Lt	3 cm	Post. to QP	+	Class II	None
19	19 M	Normal	Rt	4.5 cm	QP level	+	Class I	None
20	24 M	Low grade glioma	Rt	4 cm	Post. to QP	+	Class II	None
21	36 M	MTS	Rt	4 cm	QP level	+	Class I	None

^]^Measurement is based on postoperative MRI brain from temporal pole at the level of middle temporal gyrus.

^Δ^QP: quadrigeminal plate.

+: no postoperative disfiguring features.

^*ϕ*^MTS: Mesial temporal sclerosis.

**Table 2:** Demonstrate a features comparison among groups I and II.

Variable	Group I	Group II
Number	21	17
Mean age	28	23
Operative time (average)	3 hours and 20 minutes	3 hours and 40 minutes
Surgical opening time	15 to 25 minutes	Not calculated
Average estimated blood loss	190 mL	280 mL
Average hospital stay time	4 days	4.5 days
Cosmetic effect	No disfiguring feature	Four patients with anterior temporal depression
Chronic postcraniotomy pain	None	Three patients
Extent of posterior hippocampus resection	Posterior or at the level of quadrigeminal plate	Posterior or at the level of quadrigeminal plate
Surgical complications	Transient limitation of mouth opening ability and focal alopecia	Superficial wound infection and transient partial third nerve palsy
